# A Transparent Soil Experiment to Investigate the Influence of Arrangement and Connecting Beams on the Pile–Soil Interaction of Micropile Groups

**DOI:** 10.3390/s24165448

**Published:** 2024-08-22

**Authors:** Ziyi Wang, Jinqing Jia, Lihua Zhang

**Affiliations:** School of Civil Engineering, Dalian University of Technology, Dalian 116024, China; ziyiwang@mail.dlut.edu.cn (Z.W.); lihua2018@dlut.edu.cn (L.Z.)

**Keywords:** micropile group, pile–soil interaction, transparent soil, soil pressure, three-dimensional reconstruction

## Abstract

The use of a micropile group is an effective method for small and medium-sized slope management. However, there is limited research on the pile–soil interaction mechanism of micropile groups. Based on transparent soil and PIV technology, a test platform for the lateral load testing of slopes was constructed, and eight groups of transparent soil slope model experiments were performed. The changes in soil pressure and pile top displacement at the top of the piles during lateral loading were obtained. We scanned and photographed the slope, and obtained the deformation characteristics of the soil interior based on particle image velocimetry. A three-dimensional reconstruction program was developed to generate the displacement isosurface behind the pile. The impacts of various arrangement patterns and connecting beams on the deformation attributes and pile–soil interaction mechanism were explored, and the pile–soil interaction model of group piles was summarized. The results show that the front piles in a staggered arrangement bore more lateral thrust, and the distribution of soil pressure on each row of piles was more uniform. The connecting beams enhanced the overall stiffness of the pile group, reduced pile displacement, facilitated coordinated deformation of the pile group, and enhanced the anti-sliding effect of the pile–soil composite structure.

## 1. Introduction

As an effective method for landslide control, micropile groups (MPGs) have become widely utilized in engineering in recent years [1]. Micropile reinforcement technology has the advantages of rapid construction, high adaptability with minimal site requirements, and low maintenance costs [2,3]. This approach is particularly advantageous for the prompt rescue of small and medium-sized slopes. In practical engineering applications, micropiles (MPs) are typically positioned in multiple rows and densely arranged. This arrangement effectively capitalizes on the advantages of pile–soil interactions and enhances slope stability.

The slenderness ratio of MPs typically exceeds 30. When subjected to lateral loads, the interaction mechanism between the pile and soil is different from that of large section anti-slide piles [4,5,6,7]. Current research on MPs mainly concentrates on the failure modes [8], stress characteristics [9,10], and dynamic responses [11,12] of the piles. Traditional model box tests [13], in situ tests, and centrifuge modeling tests are commonly used for model testing. Parameters such as the displacement on the slope surface, pile bending moment, and soil pressure are analyzed to understand the pile–soil interaction mechanism. To date, fruitful results have been achieved in the research of MPs and multiple rows of MPGs. However, there is a lack of research on the internal displacement field of soil under the interaction between pile groups and soil. Transparent soil model experiments offer an intuitive approach to study the deformation characteristics and failure mechanisms between structures and soil [14,15,16] and help avoid dimensional effects on soil deformation in traditional measurement methods [17,18]. Transparent soil model tests have been successfully applied to study the soil arching effect in anti-slide piles [19], slope stability under settlement conditions [20], and morphological evolution of slope shear zones [21].

Compared to conventional anti-slip piles [22,23,24], MPs have relatively large slenderness ratios and experience greater deformation in the pile body when subjected to landslide thrust [25]. As a result, MPs often appear as multiple rows of densely spaced pile groups. The dense arrangement of pile groups facilitates the formation of composite structures between the piles and the soil. MPGs rely more on the pile–soil composite structure to support the slopes, so the mechanical characteristics of MPGs and interaction with the soil are more complex with more influencing factors. In addition, MPs have a different mechanism of pile–soil interaction from traditional anti-slip piles [26,27]. However, in mainstream design, simplified calculations of MPG are based on traditional piles [28] or soil nails [29]. Overall, most studies on MPGs concentrate on the mechanical properties of the piles or reinforcement effect of pile groups on the slopes, and there is limited exploration into the pile–soil interaction mechanism of pile groups. Therefore, an experimental method that integrates transparent soil technology with particle image velocimetry (PIV) was used to investigate the pile–soil interaction mechanisms of MPGs. Using non-contact measurement methods, the displacement of the piles and internal soil displacement changes during loading were obtained. The displacement of the soil behind the pile considering spatial effects was obtained via scanning and shooting.

In this study, the effects of pile group arrangement patterns and connecting beams on the pile group mechanism were examined by modeling slopes that were reinforced by eight groups of MPGs. PIV was used to record the lateral displacement of the pile cap, deformation field, and shear strain of the slope soil under lateral loading. Thin-film pressure sensors (FSR) were used to measure the soil pressure in front of and behind the piles. A three-dimensional reconstruction program was developed to obtain the displacement isosurface of the soil behind the piles. The pile–soil interaction and coordinated deformation mechanism of the pile group were analyzed. Various pile–soil interaction models under different arrangement configurations were summarized. The experimental results provide a basic understanding of the pile–soil composite structure of MPGs and serve as a reference for engineering applications.

## 2. Physical Model Experiment

### 2.1. Experimental Apparatus

**Figure 1:**
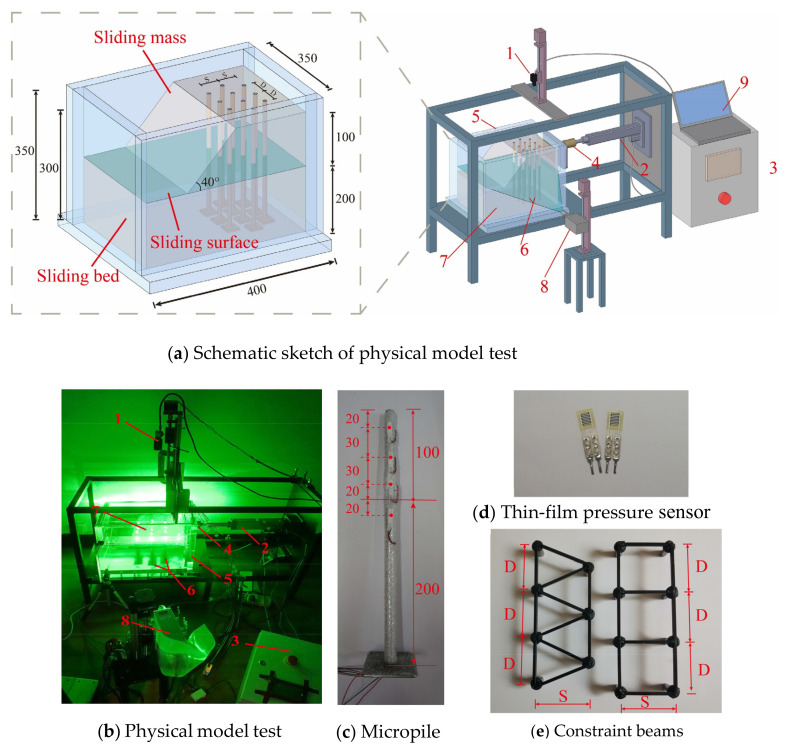
Physical model test: 1—camera; 2—loading device; 3—PLC electrical box; 4—load cell; 5—acrylic model box; 6—model pills; 7—transparent soil; 8—laser device; 9—image acquisition system.

Combining the technical features of particle image velocimetry (PIV) and model testing [30], a test platform was designed and constructed (Figure 1a,b). It included a loading system and a three-dimensional particle image velocimetry system (PIV). As shown in Figure 1, the test platform was centered on a model box. Considering the transparency and pressure bearing capacity, the model box was made of a 3 cm thick acrylic vacuum box with internal dimensions of 40 × 29 × 35 cm. On the right side of the model box was the loading system, which mainly consisted of a servo motor, a load board, pressure sensors, and a PLC electrical box. The front of the model box was the laser emission system in the PIV system, which included a 2000 mW sheet laser, an optical platform, and a high-precision linear guide rail. The image acquisition system in the PIV system was positioned above the model box and consisted of a high-speed camera with a resolution of 3000 × 4000 pixels, a high-precision linear guide rail, and an image acquisition computer. The laser and high-speed camera were fixed on the high-precision linear guide rail. Both were controlled by a dual-module controller to simultaneously move with equidistant synchronous motion and capture the light spot images of soil particles in each soil layer.

### 2.2. Transparent Soil

**Figure 2:**
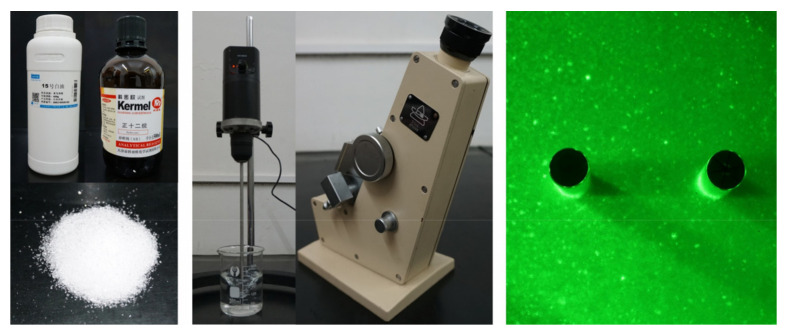
Transparent soil production.

In this experiment, the transparent soil used to construct the slope models comprised fused quartz sand and pore fluid. To satisfy the transparency and speckle field clarity requirements, fused quartz sand with a diameter of 0.5–1 mm was selected. At 25 °C, a pore fluid with a refractive index of 1.4585 was prepared using n-dodecane and #15 white oil in a mass ratio of 1:20. Figure 1a shows the fundamental dimensions of the transparent soil slope. The slope model construction process began by introducing pore fluid into the model box and placing MPGs. Then, a 5 cm thick layer of fused quartz sand was poured in, compacted, and vacuumed. This construction sequence was repeated until the pile top was reached. The sliding bed height of the slope model was 200 mm, the sliding mass height was 100 mm, and the slope angle was 40°. Three layers of PTFE film were positioned between the sliding mass and sliding bed to act as sliding surfaces. Under laser illumination, the prepared soil could generate clear light spots, as evidenced in Figure 2.

### 2.3. Micropile Group Model

Based on the specific engineering background [1] and large in situ tests [8], the similarity relationship between the proposed model and the original model was established. According to relevant specifications for micropiles [29], the slenderness ratio of micropiles is greater than 30. In this experiment, a 6063 hollow aluminum tube with a wall thickness of 0.5 mm was selected as the model pile. A steel plate was affixed to the base to enhance the resistance to the pullout force of the pile body (Figure 1c). To enhance the surface roughness of the piles, epoxy resin was used to adhere 0.5–1.0 mm fused quartz sand to the pile surface. A flat-head pin with a diameter slightly smaller than the pile diameter was used, and a 3 × 3 mm square steel bar was welded to the top of the nail to serve as a connecting beam for the pile top (Figure 1e). Black matte paint was sprayed on the surface of the beams to prevent laser reflection from interfering with image acquisition during the test. Finally, steel adhesive was applied to the side of the dowel, which was inserted into the pile to form MPGs with a connecting beam.

**Figure 3:**
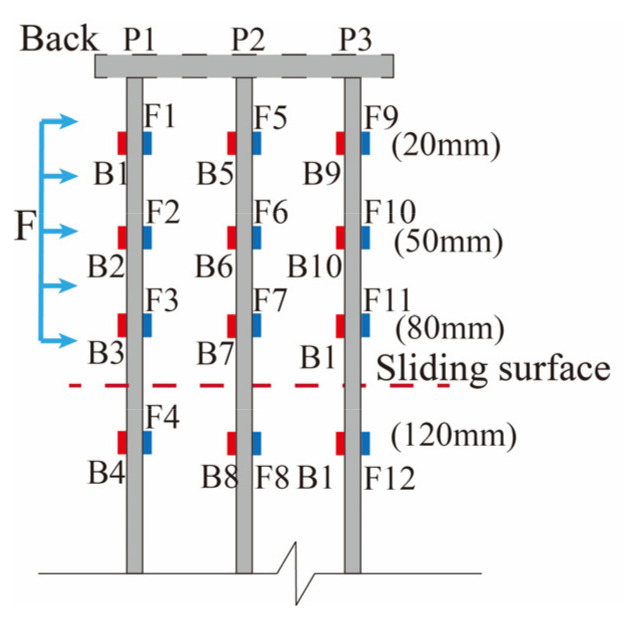
Layout diagram of FSR.

Due to the fact that a conventional soil pressure box occupies a large space and causes strong light obstruction, it affects both the laser irradiation and the collection of soil particle speckles, consequently impacting the test results. Therefore, thin-film pressure sensors [31,32] (FSRs) (Figure 1d) were utilized. They were placed 20 mm, 50 mm, and 80 mm above the sliding surface and 20 mm below the sliding surface (Figure 3) to measure the soil pressure on the MP during the loading process.

### 2.4. Testing Procedure

Based on previous experiments, spacing between piles of 5 times the pile diameter provides effective soil shielding and significantly enhances the soil arching effect behind the piles. In this experiment, the pile spacing D and row spacing S in each group were 5 times the pile diameter. Eight lateral loading tests were conducted on slope models reinforced with two or three rows of pile groups. The influence of the arrangement and connecting beams on the pile–soil effect of MPGs was studied. Table 1 shows the experimental conditions.

Before commencing the formal experiment, the laser and camera positions were adjusted to ensure that the horizontal laser plane and camera lens were perpendicular to the laser plane. This adjustment ensured that the laser plane fully illuminated the entire soil layer, and the high-speed camera captured the complete surface of the soil with clear light spots. During the loading process, the lateral panels were removed, and a 1 mm layer was applied in a single pass at a velocity of 0.5 mm/s. After each loading, the linear guide rail was controlled in steps of 2 mm to achieve equidistant synchronous movement of the laser and high-speed camera. Image data of the slope soil and pile group were captured across 50 different sections.

## 3. Results and Analysis

### 3.1. Deformation of Piles

**Figure 4:**
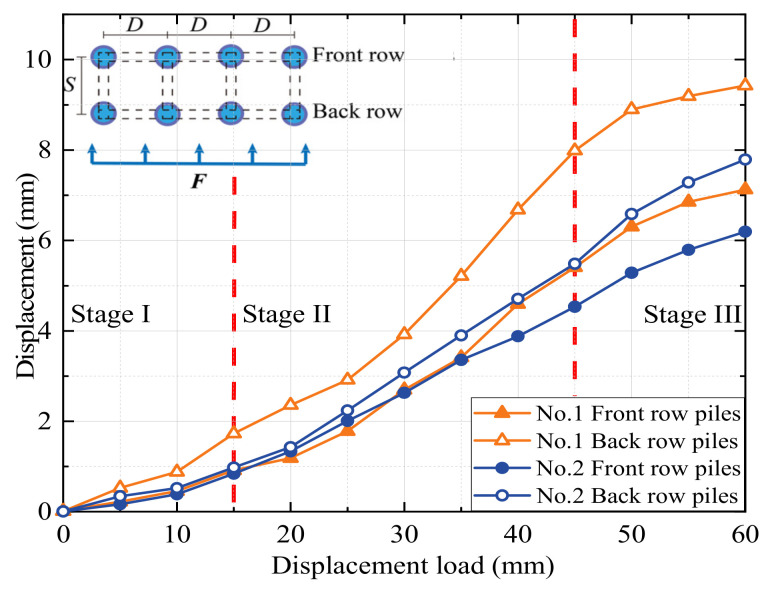
Displacement of the double-row piles in the row arrangement.

**Figure 5:**
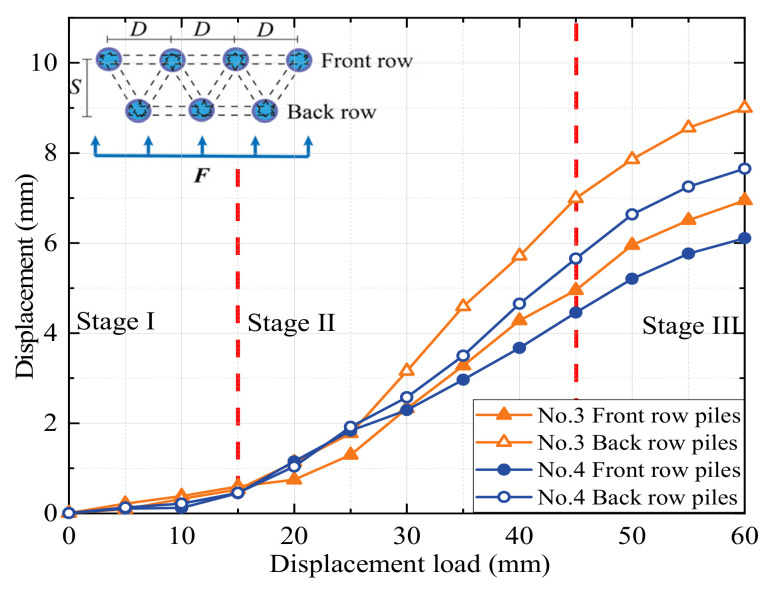
Displacement of the double-row piles in the staggered arrangement.

**Figure 6:**
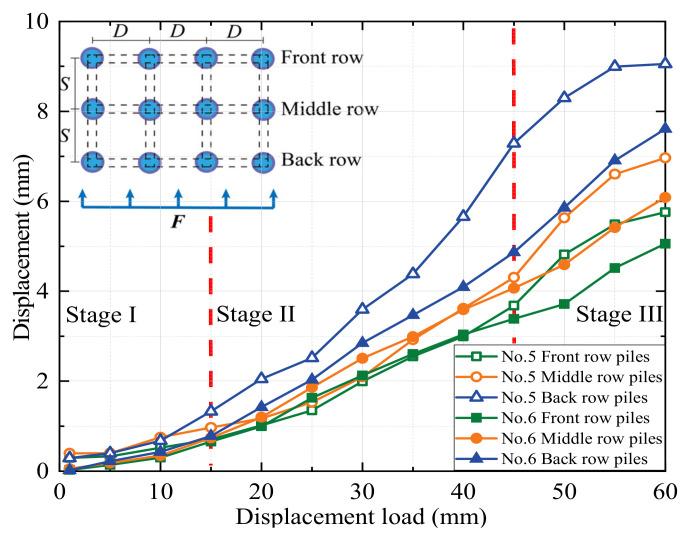
Displacement of the three-row piles in the row arrangement.

**Figure 7:**
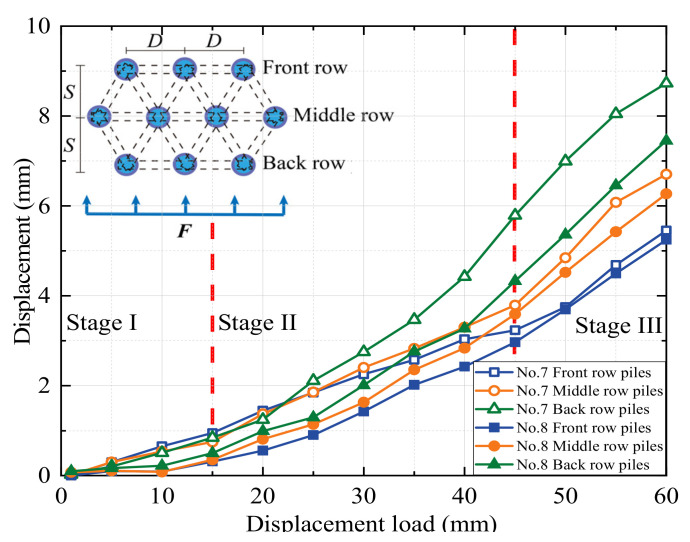
Displacement of the three-row piles in the staggered arrangement.

The images at the pile top position were analyzed using PIV technology to derive the load–settlement curves for each experiment. The PIV technique was used to obtain the deformation data of the soil by tracking the deformation process of the soil speckle field. Then, load–displacement curves of the top of the pile in each set of tests were obtained. The displacement–load curves for the pile tops of the eight modeled MPGs are shown in Figure 4, Figure 5, Figure 6 and Figure 7.

In the experiments involving specimens No. 1, No. 3, No. 5, and No. 7 without connecting beams, the distribution of landslide thrusts across each row varied. The back-row piles exhibited the highest displacement curve rate, which gradually decreased for the middle and front rows. The load–settlement curve at the pile tops revealed a noticeable settlement trend and could be roughly divided into three stages. In stage I (displacements of 0–15 mm), the deformation was relatively slow, with less than 1 mm displacement for most piles, except the back-row piles of experiment No. 1. In stage II (displacements of 15–45 mm), the displacement deformation accelerated, and the curve rate significantly increased. The deformation rate of the back-row piles surpassed that of the front-row piles. In group No. 3, the displacement increased by 4.4 mm for the front piles and 6.5 mm for the back piles. In stage III, when the load displacement was 45–60 mm, the displacement curve of the back-row piles tended to stabilize, and the displacement growth of the front-row piles slowed.

In experiments No. 2, No. 4, No. 6, and No. 8 with connecting beams, the displacement was transmitted through the connecting beams. The coordinated deformation of the pile group resulted in small displacement differences among the rows of piles. Under the same arrangement, the pile groups with connecting beams displaced less than those without connecting beams. The greater stiffness of the connecting beams resulted in cooperative deformation between the rows of piles under their constraint effect. The connecting beams enhanced the overall stiffness of the pile group and its effectiveness in soil restraint, which decreased the rate of pile failure, the slope in the load–displacement curve at the pile, and phase changes. This result also indicates a negative correlation between pile displacement and the overall stiffness of the pile group. Greater stiffness of the pile group corresponds to smaller displacement of the piles.

When the load displacement was 45 mm, the displacements of the pile top in the front or middle rows of piles No. 3 and No. 7 in the staggered arrangement were 4.2 mm and 3.8 mm, respectively. The displacements of the pile top in the front or middle rows of No. 1 and No. 5 in the row arrangement were 4.6 mm and 4.3 mm, respectively. Due to the row pile arrangement, the front rows bore more of the soil pressure caused by the displacement of the back rows of piles. In the staggered arrangement, the front rows also bore the thrust of the soil between the piles, and the forces on each row of piles were more evenly distributed. In the pile groups with connecting beams, the concentrated force at the top of the front-row piles was greater, which resulted in the bending deformation and slight forward flipping of the pile group. When the load displacement reached approximately 15 mm, a displacement difference occurred at the top of each row of piles, and the difference increased with increasing load. When the loading displacement was 60 mm, the displacement differences between front and back rows of the pile top in experiments No. 2 and No. 4 were 1.6 mm and 1.5 mm, respectively. In experiments No. 6 and No. 8, the differences were 2.6 mm and 2.2 mm, respectively. The flipping angle of the staggered-pile-arrangement group was small due to the staggered arrangement of the pile group, where the middle and front rows of the loaded section bore greater lateral soil pressure than the lateral thrust of the pile top. To some degree, each row of piles could fully exert support in a staggered arrangement, so the staggered arrangement was more reasonable and had a better support effect.

### 3.2. Soil Pressure on Piles

**Figure 8:**
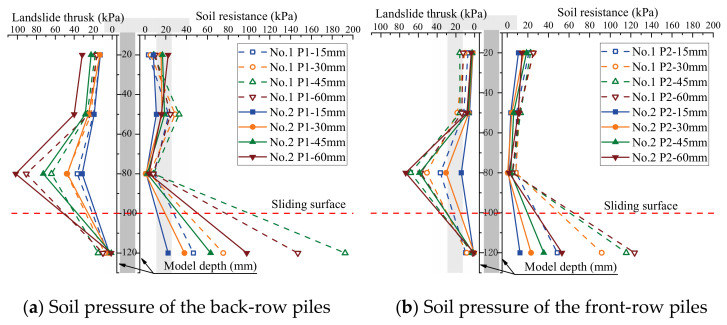
Soil pressure of the double-row-arrangement piles.

**Figure 9:**
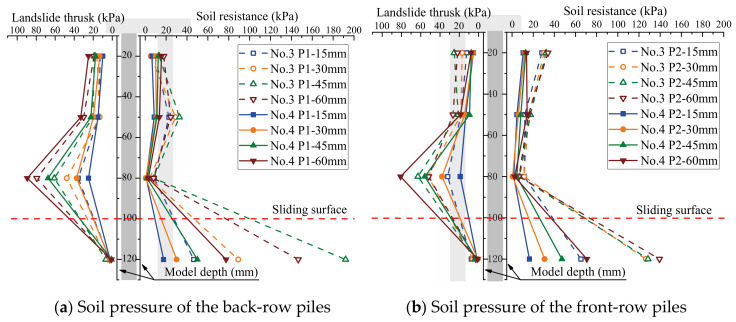
Soil pressure of the piles with a double-row staggered arrangement.

**Figure 10:**
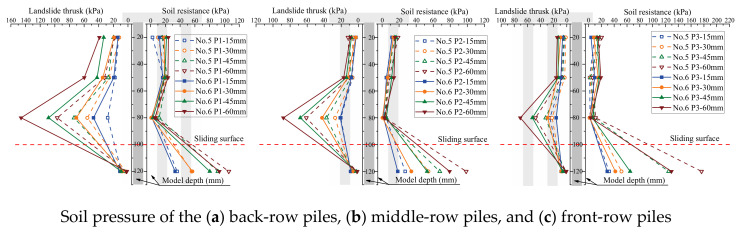
Soil pressure of the three-row pile arrangement.

**Figure 11:**
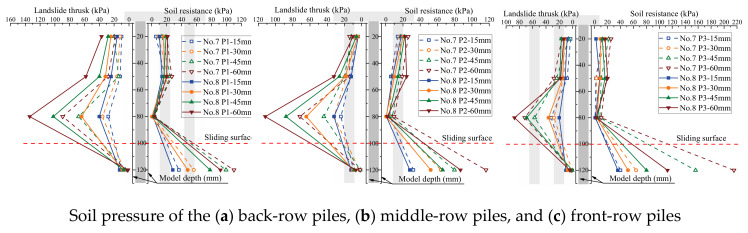
Soil pressure of the three-row staggered-arrangement piles.

The soil pressure on piles is a crucial metric to assess the bearing capacity of piles in the soil. The influence of the arrangement of the MPGs and connecting beams on the soil pressure of the piles was investigated. Three piles per row were selected to deploy the FSRs (Figure 1d). Soil pressure measurements were taken in front of and behind the piles in each row, as illustrated in Figure 4. The soil pressure data corresponding to displacement loads of 15 mm, 30 mm, 45 mm, and 60 mm are shown in Figure 8, Figure 9, Figure 10 and Figure 11.

The soil pressure behind the pile on the upper part of the sliding surface exhibited a roughly triangular distribution pattern and increased with depth. The peak soil pressure was observed at measuring points B3, B7, and B11 on the upper section of the sliding surface (Figure 8a, Figure 9a, Figure 10a and Figure 11a). This is consistent with the results of other micropile model experiments [8,33]. The embedding effect caused smaller deformation of the pile body at these measuring points compared to the displacement of the soil. Consequently, the soil pressure increased due to the compression of soil behind the pile near the sliding surface. The soil resistance beneath the sliding surface behind the pile was relatively low. When the load increased, the soil pressure at this measuring point decreased. Because the pile body experienced bending deformation, the pile body separated from the soil at the sliding bed location. Additionally, the thickly layered sliding surface in the experiments contributed to the relatively low soil pressure on the pile at this point.

The experimental data show that the soil pressure behind the pile with the connecting beam was higher. When the load increased, the soil pressures behind each row of piles had similar variation trends. Figure 8b, Figure 9b, Figure 10b and Figure 11b, and Figure 10c and Figure 11c illustrate the soil pressure behind the front and middle rows of the pile group. Under a specific displacement load, the distribution of soil pressure at the upper part of the sliding surface initially increased and subsequently decreased with depth. At a displacement load of 6 mm, the soil pressures at measurement point B7 for experiments No. 2 and No. 4 were 73.5 kPa and 80.8 kPa, respectively. These values were 16.0 kPa and 29.1 kPa higher than those at the same positions in experiments No. 1 and No. 3, respectively. Thus, the upper soil pressure of the front-row piles in the pile group with connecting beams was greater than that in the pile group without connecting beams.

When the load displacement increased from 15 mm to 45 mm, the soil pressures at measurement point B7 in experiments No. 2 and No. 4 increased by 44.7 kPa and 36.9 kPa, respectively. In contrast, those at the same position in experiments No. 1 and No. 3 increased by only 31.7 kPa and 30.9 kPa, respectively. This result indicates that the soil pressure increase was more significant for the pile group with connecting beams than that without connecting beams. The connecting beams enhanced the integrity and stiffness of the pile group, and effectively limited the displacement (Figure 4, Figure 5, Figure 6 and Figure 7) and reduced the bending deformation of the back-row piles. This behavior ensured the stability of the arch foot at the soil arch behind the piles, which resulted in higher soil pressures. In pile groups without connecting beams, load transfer could not occur between the rows of piles, which resulted in lower stiffness and greater overall stiffness of the pile group. As a result, the soil pressure behind the piles decreased, and the increase in soil pressure due to the load decreased. In summary, the soil pressure in MPGs is positively correlated with the bending stiffness of the pile group. Greater bending stiffness of the pile group corresponds to smaller displacement of the pile and greater soil pressure behind the pile.

Compared with the pile groups with the row arrangement (No. 5 and No. 6) (Figure 10c), the front-row piles of those with the staggered arrangement (No. 7 and No. 8) experienced higher soil pressure (Figure 11c). When the displacement load was 15 mm, the soil pressures at measurement point B11 of the staggered-arrangement piles were 6.41 kPa and 3.81 kPa higher. When the displacement load was 60 mm, the soil pressures at that location were 24.3 kPa and 15.4 kPa higher. The reason was that in the staggered arrangement, the soil pressure on the front-row piles was predominantly from the soil pressure between the back-row piles.

The soil pressure in front of the pile primarily indicated the resistance of the soil in front of the pile group. Above the sliding surface of the back-row piles, the soil resistance increased along the depth direction (Figure 8a, Figure 9a, Figure 10a and Figure 11a). Conversely, at the pile top, the soil resistance was greater in the middle and front rows and decreased with increasing depth. The soil pressure at measurement point F12 below the sliding surface was the largest in each group of experiments due to the larger relative displacement between the pile and soil in the anchored section compared to the anti-sliding segment. At the sliding surface, the soil had to provide higher resistance to resist the bending and shear forces from the upper structure. Additionally, the overall stiffness of the pile group intensified the pile–soil pressure at this location. When the loading displacement was 60 mm, the soil pressures at measurement point F10 in experiments No. 6 and No. 8 were 19.2 kPa and 20.3 kPa, respectively. In groups No. 5 and No. 7, the measurements at point F10 were 6.4 kPa and 18.0 kPa, respectively. Comparing Figure 8b and Figure 9b and Figure 10c and Figure 11c, under identical conditions, the front row of piles with connecting beams exhibited marginally higher soil pressure because the connecting beams induced coordinated displacement changes. Thus, the front-row piles displaced more than what the surrounding soil might induce, so the soil pressure slightly increased in the loaded segment of the piles.

### 3.3. Displacement of the Soil

**Figure 12:**
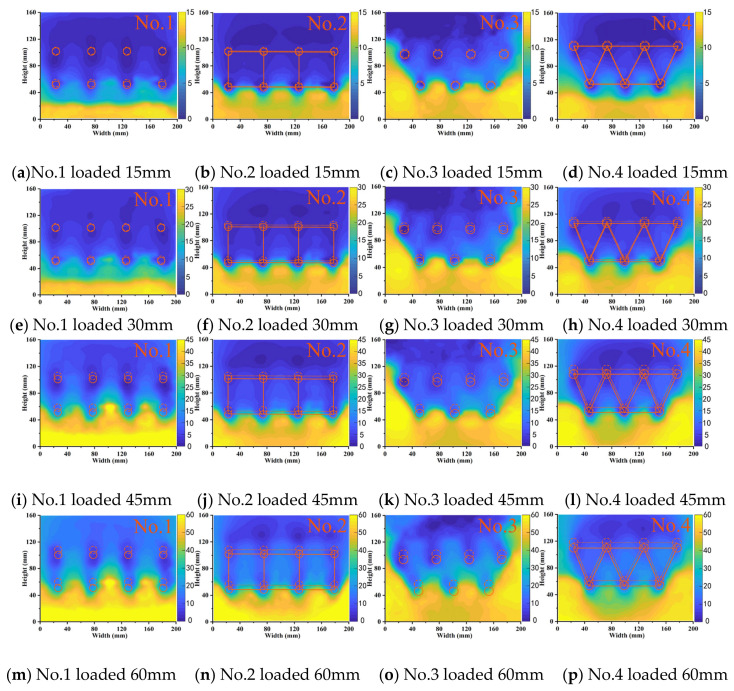
Displacement cloud images of soil of the two-row pile group.

**Figure 13:**
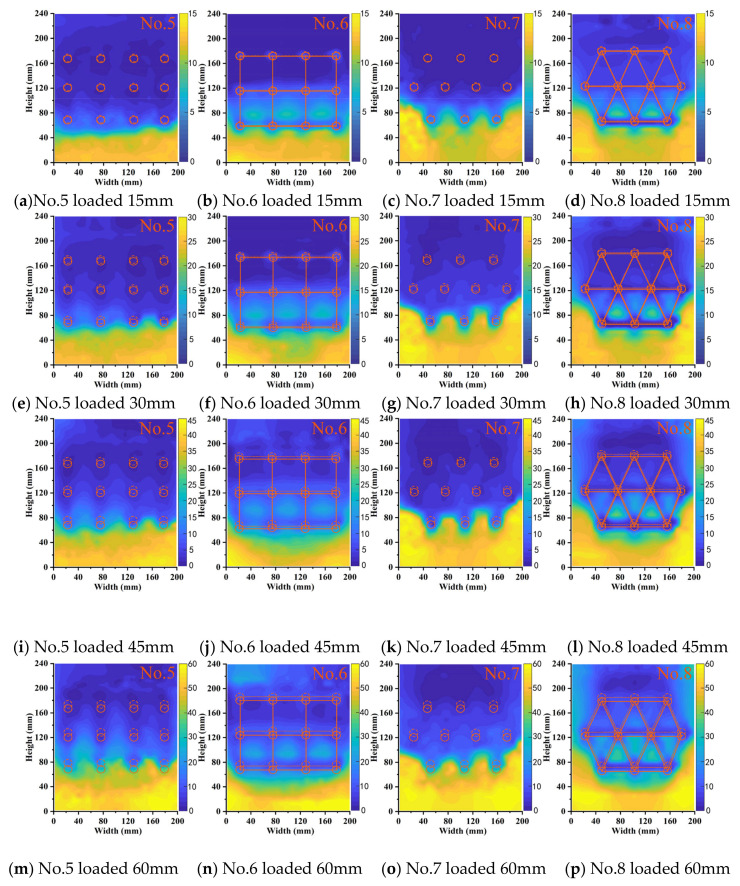
Displacement cloud images of soil in the three-row pile group.

Displacement was induced in the slope soil under lateral load application. PIV technology was used to process the captured soil light spot images and obtain a displacement cloud image of the soil. The color variations in these cloud maps indicate the extent of soil displacement. Figure 12 and Figure 13 show the displacement cloud maps of the soil at 4 cm above the sliding surface in the two-row and three-row MPG experiments, respectively. The loading displacements for the four rows were 15 mm, 30 mm, 45 mm, and 60 mm. The solid lines in the displacement cloud images denote the initial positions of the pile groups and connecting beams, and the dashed lines indicate their post-loading positions.

In the initial loading stage (Figure 12a–d), the back-row piles had similar shielding effects on the soil and formed relatively stable soil arches. The displacement of the pile group was small, and the differences between the groups were not significant. However, because there were fewer back-row piles in the staggered arrangement, some soil slid around the pile groups from the sides. In the middle stage, the loading displacements were 15–45 mm (Figure 12e–l). The soil arch behind the back piles gradually thinned and ultimately failed, which resulted in a notable increase in soil displacement between the piles. In this stage, the front-row piles in the row-arrangement piles only relied on frictional arching, so there was substantial soil displacement between the piles. In contrast, the front-row piles in the staggered arrangement of pile groups provided better shelter for the soil between the piles. In the final loading stage (Figure 12m–p), the soil behind the piles in the no-connecting-beam experiments was completely compromised, so soil flowed out from between the piles.

Figure 13 shows the displacement cloud images for the three-row micropile groups (MPGs). The reinforcement effect of three rows of MPGs on the soil was better than that in Figure 12. No. 5 and No. 7 were the experiments without connecting beams. In experiment No. 7, the piles were in a staggered arrangement. The front row of piles blocked the remaining landslide thrust between the piles in the latter row. Each row of piles could simultaneously bear the landslide thrust. Despite numerous piles in experiment No. 5, there was no marked difference in displacement between the two experiments. No. 6 and No. 8 were the experiments with connecting beams. The displacement of the soil surrounding the first two rows of piles in both groups was greater than that without a connecting beam in the same arrangement. This increase is attributed to the connecting beams that integrated the pile groups. There was a transfer of displacement between the pile groups. The piles in the front two rows were subjected to the thrust of the connecting beams, which resulted in slightly larger displacement of soil in front of the piles.

### 3.4. Shear Strain of the Soil

**Figure 14:**
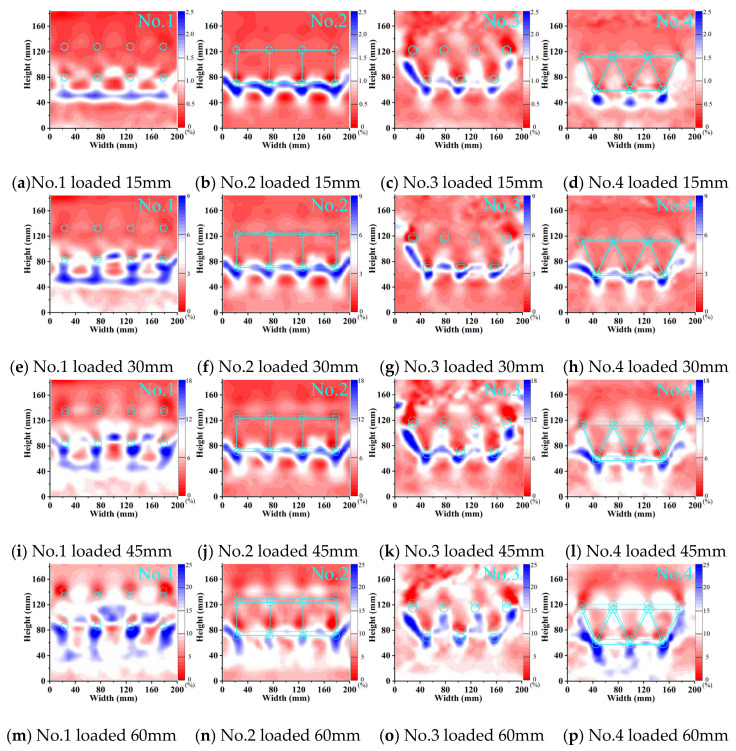
Shear strain of the two-row pile group.

**Figure 15:**
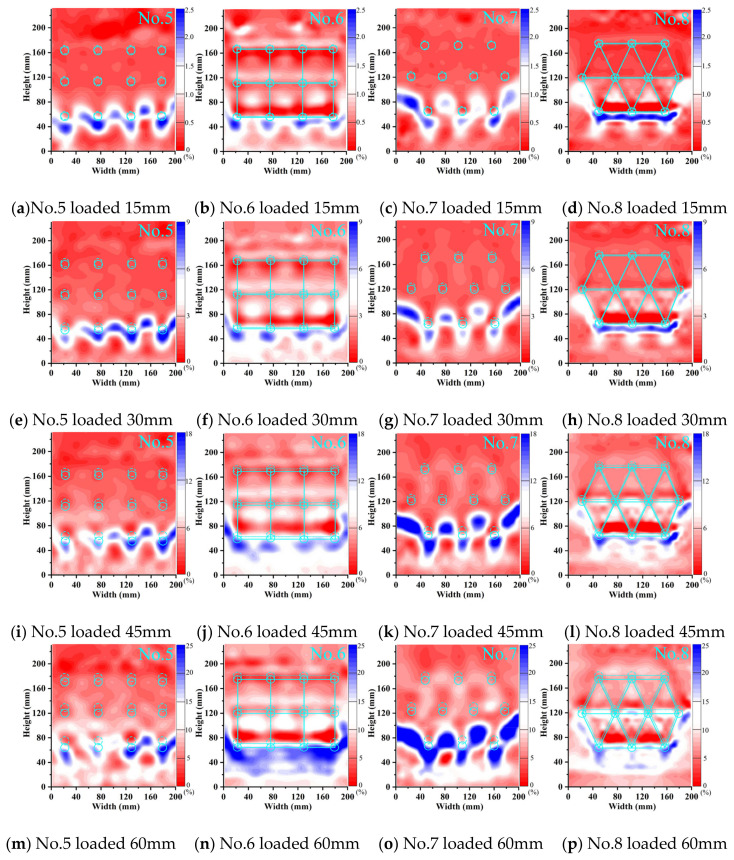
Shear strain of the three-row pile group.

The stress transfer phenomenon in soil arching behind the pile was obtained through the shear strength of the soil. In the shear strain cloud image, this behavior manifested as an arched ring with a specific thickness. Figure 14 and Figure 15 show the shear strain cloud images for double-row and three-row MPGs under loading displacements of 15–60 mm. These figures show the conversion between stationary and loaded sections.

During the initial loading stage, arching shear bands were observed behind the back-row piles in each double-row pile foundation experiment. In the middle and late stages of loading, a clear arched shear zone formed behind the front piles in the experiments without connecting beams. With increasing loads, the shear strain of the soil behind each row of piles intensified and expanded in scope. Compared to the experiments without connecting beams, the shear strain of the soil behind the front-row piles in the experiments with connecting beams slightly decreased. When the load increased, the soil shear stress in front of the front-row piles only slightly increased. This phenomenon occurred due to the synchronized deformation of the pile group facilitated by the connecting beams. The front pile exhibited reverse relative displacement with the piles and soil, which was induced by the concentrated force from the connecting beam. Under this displacement, the shielding effect of the pile on the soil behind the pile decreased, and the soil in front of the pile exerted a resisting force on the pile.

In the three-row pile groups, the stress characteristics and failure process of each row of piles were not uniform (Figure 15). With increasing load, the shear stress of the soil gradually developed from the back row to the front row with obvious progressive failure characteristics. The formation and development process of shear bands around the pile under different arrangement patterns could be observed to compare and analyze the pile–soil interaction relationship of the pile groups. During the initial loading stage, the back and middle rows of piles in the staggered arrangement collectively resisted the landslide force. Significant shear deformation occurred in the soil behind the piles in both rows. In the middle and late loading stages, the positions of the shear bands and values of shear strain in the middle and front rows of the pile–soil system were different for the two arrangement patterns. In the row arrangement, the largest shear force of the group pile appeared between the two piles, and the maximum shear force appeared at the crown of the arch. In the staggered arrangement, the shear force of the soil behind the pile was greater and connected to the pile, and the maximum shear force occurred at the arch foot. With a staggered arrangement, the front row of piles had a good shielding effect on the soil between the piles. It directly participated in the stress transfer between the piles and soil, which formed distinct arching shear bands. In the staggered arrangement, the pile group had both friction arches and end bearing arches behind the piles. In the row arrangement, the front piles only bore the soil pressure caused by the displacement and deformation of the back-row piles. The friction arch on the side of the pile played a greater role.

### 3.5. Three-Dimensional Reconstruction

**Figure 16:**
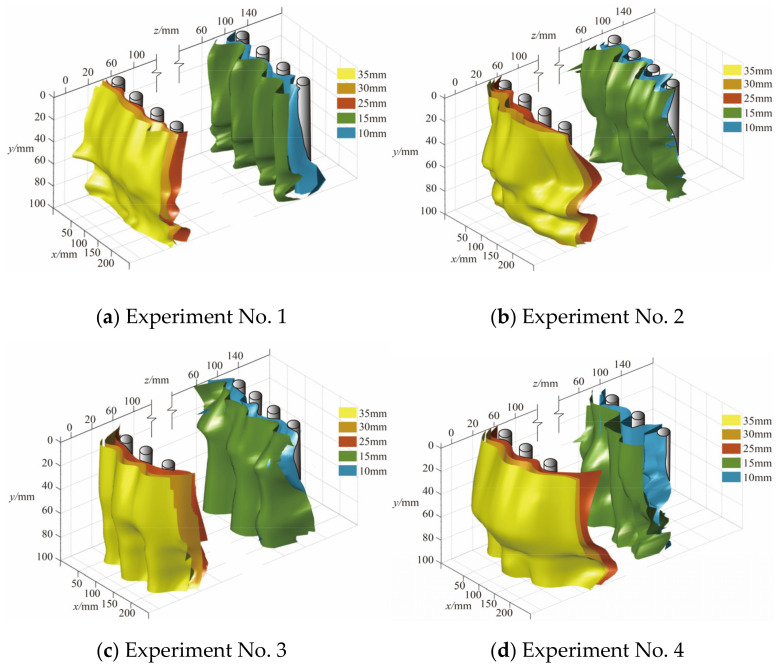
Three-dimensional reconstruction of soil isosurfaces behind two rows of piles.

**Figure 17:**
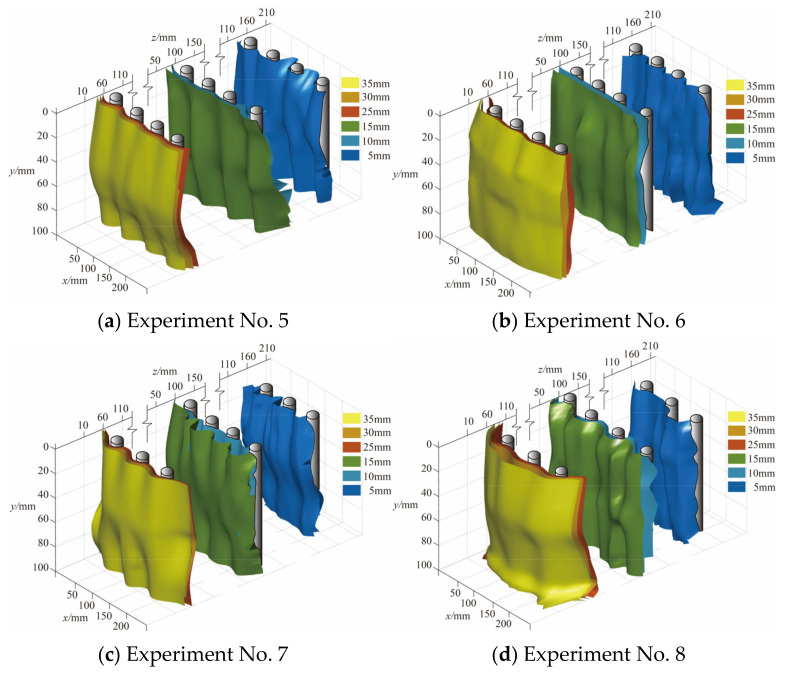
Three-dimensional reconstruction of soil isosurfaces behind the three-row piles.

During the loading process, a “CT scan” was performed on the slope, and deformation cloud images of the soil at each section were obtained. Isolines representing equal displacements in these cloud maps were extracted and smoothed. The cubic spline difference function was adopted, and a linear difference algorithm was applied to the contours of the same displacement in each layer to smooth the resulting three-dimensional reconstructed isosurface. Finally, the “fill outliers” function was used to detect and replace outliers to improve the reliability and accuracy of the data. Figure 16 and Figure 17 show the 3D reconstruction results of the soil behind the piles of the two-row and three-row pile groups when the loading displacement was 45 mm. Despite potential inaccuracies in the z-direction soil displacement, these data provided reasonable information on the deformation trends behind each row of piles.

The isosurface behind the pile increased with depth, and the thickness of the soil arch first increased and subsequently decreased. The pile–soil arch mainly appeared in the central and deep soil near the slip surface. In the double-row pile groups, the maximum thickness of the isosurface of the front piles was near the pile top. In the three-row pile group, the isosurface of the middle row of piles was similar to that of the front piles of the two-row pile group. The isosurface of the front pile was relatively smooth and did not significantly change with depth, because MPs are prone to significant deformation when subjected to landslide thrusts. The upper shallow soil layer could not provide stable arch support due to the significant displacement of the pile, so the soil arch structure thinned or failed. The maximum soil arch positions of experiment Nos. 2–8 with contact beams were 35–55 mm, 30–55 mm, 35–55 mm, and 20–45 mm, respectively. The maximum location of the soil arch was closer to the top of the pile than that of the experimental group without linked beams in the same arrangement. The reason is that the connecting beam increased the stiffness of the pile group and reduced the displacement of the pile top. It could provide stable arch footing for the soil arch behind the pile, which made the maximum soil arch closer to the pile top. In the staggered arrangement, the 3D isosurface of the back or middle row of piles was thicker. In this arrangement, the front piles bore the soil pressure caused by the displacement of the back-row piles and bore the lateral load of the soil between the row piles. The pile group had a better barrier effect, and it could bear more loads.

## 4. Discussion

In practical engineering, pile groups are typically arranged [34] due to the small diameter of MPs. The pile and soil arching effect in the group serve to resist landslide thrust. In contrast to traditional anti-slide piles, MPs are prone to large deformations during loading, and the interaction between the pile and soil is complex. In this article, PIV technology [15] was used to directly observe the displacement of soil in eight transparent soil model experiments. The effect of the arrangement of the pile groups and connecting beams on the anti-sliding mechanism was investigated. In general, the support effect of pile groups with a staggered arrangement of contact beams is better, and the support effect of three rows of group piles is better than two rows of group piles. However, blindly increasing the number of rows of group piles does not have much effect on the enhancement of the support effect, and reduces the economic benefits.

### 4.1. Comparative Analysis with Previous Research

Due to the limitation of soil transparency, the slope model was simplified in this experiment. Established model box tests were referenced [35] for the horizontal loading of micropile-reinforced slopes. The pile–soil interaction mechanism was mainly investigated, thus simplifying the effect of the vertical component of landslide thrust on the group of piles. The test results showed that the laws of pile top displacement, soil pressure along the depth, and group pile failure mode were consistent with the existing model box tests [35] and large-scale field tests [8]. Additionally, among the eight experiments conducted with transparent soil, similar variation trends were observed, which to some extent validates the reliability of our results. Furthermore, the data for soil pressure along the pile body and pile head displacement were obtained by averaging the measurements from the same row of piles to minimize experimental errors.

### 4.2. Effect of the Arrangement

**Figure 18:**
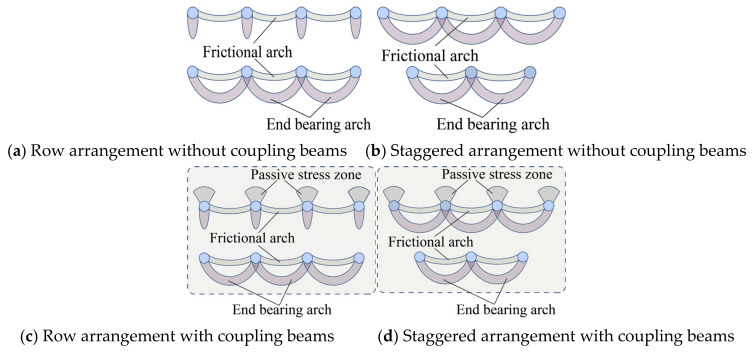
Pile–soil interaction model of the micropile group.

The essence of the earth arch effect is the transfer of earth pressure from the yield zone to the relatively static zone [36]. Many researchers attribute the soil arching effect to the non-uniform deformation of the soil [37]. In the context of soil arching between the piles of a pile group, the relatively stable zones are the sides and backs of the piles, and the yielding zones are the soils situated between the piles. Given the high slenderness ratio of micropiles, significant displacement occurs under substantial lateral loads, so the soil arch loses its stable footing. Longitudinally, the displacement at the pile top is more pronounced, so the maximum thickness of the soil arch behind the micropile shifts closer to the sliding surface (Figure 17 and Figure 18).

Under lateral thrust, the soil between the piles exhibited uneven displacement (Figure 13 and Figure 14) and stress redistribution (Figure 15 and Figure 16). The non-synchronous development of soil arches between piles in each row was influenced by the arrangement pattern. The soil pressure and displacement on the back-row pile were the greatest, and the highest position of the soil arch on the isosurface behind the pile was in the central and deep parts. The row arrangement reduced the displacement of the front-row piles and soil pressure behind them. Figure 18a shows a pile group soil arch schematic for the row arrangement of the MPGs. Friction arches and end bearing arches formed between the piles in the back row. The soil pressure behind the front piles showed minimal sensitivity to changes in displacement load. Based on the shear strain cloud image development process of the three-row pile group arrangement (Figure 16a–m), we speculated that the front row was dominated by inter-pile friction arches. With increasing displacement of the back row (Figure 5 and Figure 7) and the resultant deformation thrust, a range of pressure zones developed behind the front-row piles.

Figure 18b shows the soil arch effect in a pile group that featured staggered MPGs. The soil arch effect of the back row of piles was similar for both arrangement patterns. The front piles bore the thrust of the soil between the back piles, which resulted in large displacement and large soil pressure behind the piles. A staggered configuration can enhance the anti-sliding performance of the leading piles, make the distribution of soil pressure in each row more uniform, and enhance the overall effect of the pile group.

### 4.3. Effect of the Connecting Beams

In soil landslides, micropiles frequently experience plastic failure due to significant deformation [38]. Therefore, reducing the displacement of MPs is a method to prevent bending damage to the pile group. In practical engineering, the connecting beam has much greater stiffness than MPs [39]. Under lateral loading, connecting beams facilitate coordinated deformation, which effectively enhances the stiffness of the MPG, reduces the displacement of the pile shaft, and ensures the stability of the soil arch at the foot of the piles. This behavior enhances the stability of the pile–soil composite structure, bending resistance of the pile group, and soil pressure behind the piles.

Figure 18c,d show a schematic diagram of the pile group soil arch with the connecting beam MPG. Due to the large slenderness ratio of the micropile, a large amount of lateral deformation was generated under the landslide thrust. The top of the front pile was displaced more under the concentrated force of the connecting beam than the displacement caused by soil around the pile. Thus, a passive stress zone formed in the soil in front of the piles [40,41]. Additionally, the stiffness of the connecting beams significantly exceeded that of the piles, which implies that the pile spacing remains constant during the loading process [39]. The deformation thrust in the back row due to the increased displacement hardly affected the front pile. Under the condition of contact beams, the area behind the front row of the piles did not develop an extensive area of compression. In the staggered arrangement, the front piles strongly shielded the soil. The development of the arch between the front piles is higher than that in the row arrangement [8].

### 4.4. Limitations and Further Work

This article intends to explore the reinforcement impact and pile–soil interaction of micropile groups in various arrangement patterns and the role of the connecting beams. Due to the limitations of the visual depth of the soil and size effect of the micropile, it is difficult to establish an overall slope model. However, the limited visibility into the transparent soil depth and size effect of the micropile made establishing a comprehensive slope model challenging. Additionally, this investigation only considered horizontal loads on the MPGs. Therefore, it would be meaningful to assess the impact of the landslide thrust angle on MPG reinforcement effectiveness and the pile–soil interaction mechanism in future studies.

## 5. Conclusions

In this study, the behavior of micropile groups under lateral loading and the mechanism of pile–soil interaction were studied through transparent soil model experiments. The effects of the pile group arrangement and connecting beams were analyzed and discussed. The main conclusions are summarized as follows:

1. The soil pressure behind the pile forms a triangular distribution, and the highest pressure is near the sliding surface. This pressure is directly correlated with the overall bending stiffness of the pile group.

2. The connecting beams increase the overall rigidity of the pile group and reduce the pile group displacement. This effect results in a stable arch foot for the soil arch behind the pile and promotes the formation of a stable pile–soil composite structure.

3. The middle and front rows bear heavier loads in staggered arrangements than in row arrangements. However, the effectiveness of the middle and front rows is limited in the row arrangement configuration.

4. Through 3D reconstruction, a pattern of soil displacement with depth variation can be visually observed. The highest point of the isosurface behind the micropiles is near the sliding surface. And the greater the stiffness of the pile group, the closer this position is to the pile top.

## Figures and Tables

**Table 1:** Specimen design details.

Table	Experiments	Arrangement Form	Coupling Beam	Sketch
Group 1 Double rows	No.1	Row	Without coupling beam	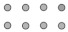
	No.2	Row	With coupling beam	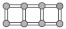
	No.3	Staggered	Without coupling beam	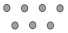
	No.4	Staggered	With coupling beam	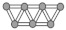
Group 2 Three rows	No.5	Row	Without coupling beam	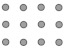
	No.6	Row	With coupling beam	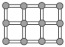
	No.7	Staggered	Without coupling beam	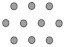
	No.8	Staggered	With coupling beam	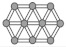

## Data Availability

The original contributions presented in the study are included in the article, further inquiries can be directed to the corresponding author.

## References

[1] Zhang Y., Lei Y., Qiang X., Wu D., Wang D., Wang J. (2023). Centrifugal model test of slope reinforced by multi-row micro-pile frame structure. Rock Soil Mech..

[2] Aboutabikh M., Soliman A.M., El Naggar M.H. (2020). Performance of hollow bar micropiles using green grout incorporating treated oil sand waste. J. Build. Eng..

[3] Wang C.C., Han J.T., Kim S. (2022). A field study on the load sharing behavior of a micropiled raft underpinned by a waveform micropile. Can. Geotech. J..

[4] Sun Z.-J., Wang Q., Min R., Duan Q.-W. (2023). Sensitivity analysis of influencing factors of pile foundation stability based on field experiment. Structures.

[5] Gong W., Tang H., Juang C.H., Wang L. (2020). Optimization design of stabilizing piles in slopes considering spatial variability. Acta Geotech..

[6] Gong W., Tang H., Wang H., Wang X., Juang C.H. (2019). Probabilistic analysis and design of stabilizing piles in slope considering stratigraphic uncertainty. Eng. Geol..

[7] Pei Z.W., Zhang Y., Nian T., Song X., Zhao W. (2023). Performance investigation of micropile groups in stabilizing unstable talus slopes via centrifuge model tests. Can. Geotech. J..

[8] Liu X.L., Yan J.K., Liu L., Han B. (2021). Large-Scale Model Test of a Micropile Group for Landslide Control. Adv. Civ. Eng..

[9] Hussain Z., Sharma B., Rahman T. (2019). Micropile group behaviour subjected to lateral loading. Innov. Infrastruct. Solut..

[10] Kyung D., Lee J. (2018). Interpretative Analysis of Lateral Load-Carrying Behavior and Design Model for Inclined Single and Group Micropiles. J. Geotech. Geoenviron. Eng..

[11] Li N., Men Y.M., Yuan L.Q., Gao H., Li J., Wang B.Q. (2020). Study on the Mechanical Characteristic of Micropiles Supporting Landslide under Step-Loadings. Geotech. Geol. Eng..

[12] Dong J., Wu Z.H., Li X., Chen H.Y. (2018). Dynamic response and pile-soil interaction of a heavy-haul railway embankment slope reinforced by micro-piles. Comput. Geotech..

[13] Li N., Men Y.M., Yuan L.Q., Wang B.Q., Li J., Liu X.L. (2019). Seismic response of micropiles-reinforced landslide based on shaking table test. Geomat. Nat. Hazards Risk.

[14] Yuan B.X., Sun M., Xiong L., Luo Q.Z., Pradhan S.P., Li H.Z. (2020). Investigation of 3D deformation of transparent soil around a laterally loaded pile based on a hydraulic gradient model test. J. Build. Eng..

[15] Liu C., Tang X.W., Wei H.W., Wang P.P., Zhao H.H. (2020). Model Tests of Jacked-Pile Penetration into Sand Using Transparent Soil and Incremental Particle Image Velocimetry. Ksce J. Civ. Eng..

[16] Wang Z.T., Luo G.Y., Kong G.Q., Zhang Y., Lu J.Q., Chen Y., Yang Q. (2022). Centrifuge model tests on anchor pile of single point mooring system under oblique pullout load using transparent sand. Ocean. Eng..

[17] Wang J.X., Liu X.T., Liu S.L., Zhu Y.F., Pan W.Q., Zhou J. (2019). Physical model test of transparent soil on coupling effect of cut-off wall and pumping wells during foundation pit dewatering. Acta Geotech..

[18] Li Y.Z., Zhou H., Liu H.L., Ding X.M., Zhang W.G. (2021). Geotechnical properties of 3D-printed transparent granular soil. Acta Geotech..

[19] Chen Q., Dong G., Wang C., Zhu B., Zhao X. (2020). Characteristics Analysis of Soil Arching Effect Behind Pile Based on Transparent Soil Technology. J. Southwest Jiaotong Univ..

[20] Sui W.H., Zheng G.S. (2018). An experimental investigation on slope stability under drawdown conditions using transparent soils. Bull. Eng. Geol. Environ..

[21] Wei L.T., Qiang X., Wang S.Y., Wang C.L., Xu J. (2020). The morphology evolution of the shear band in slope: Insights from physical modelling using transparent soil. Bull. Eng. Geol. Environ..

[22] Yang C., Tong X., Wu D., Lian J., Ding X. (2023). A new model for mechanical calculation of h-type anti-slide piles. Structures.

[23] Hu X.L., Liu D.Z., Niu L.F., Liu C., Wang X., Fu R. (2021). Development of soil-pile interactions and failure mechanisms in a pile-reinforced landslide. Eng. Geol..

[24] He C., Hu X.L., Liu D.Z., Xu C., Wu S.S., Wang X., Zhang H. (2020). Model tests of the evolutionary process and failure mechanism of a pile-reinforced landslide under two different reservoir conditions. Eng. Geol..

[25] Jia J., Wang Z. (2022). Kinematic limit analysis method for micro-pile reinforced 3D trapezoidal slope. Arab. J. Geosci..

[26] Pei Z., Zhang Y., Nian T., Xiao S., Liu H. (2023). Cross-Scale Analysis on the Working Performance of Micropile Group and Talus Slope System. Sustainability.

[27] Li C.D., Wu J.J., Tang H.M., Wang J., Chen F., Liang D.M. (2015). A novel optimal plane arrangement of stabilizing piles based on soil arching effect and stability limit for 3D colluvial landslides. Eng. Geol..

[28] Federal Highway Administration (2000). Micropile Design and Construction Guidelines Implementation Manual.

[29] Federal Highway Administration (2005). Micropile Design and Construction.

[30] Liu C., Tang X.W., Wei H.W., Zhao H.H. (2021). A New Spatial Deformation Measurement Method Using 3D Reconstruction Technology during Pile Penetration. Ksce J. Civ. Eng..

[31] Liu K., Xu C., Jia K., Zhang X. (2020). Measurement of earth pressures on curved surface of thin film pressure sensor. Chin. J. Geotech. Eng..

[32] Dontha B., Swearingen K., Swearingen S., Thrane S.E., Kiourti A. (2022). Wearable Sensors Based on Force-Sensitive Resistors for Touch-Based Collaborative Digital Gaming. Sensors.

[33] Liu X.L., Liu Y.S., Liu K., Su Y.Y. (2022). Experimental investigation on anti-sliding performance of grouted micro-pipe pile groups. Nat. Hazards.

[34] Moayed R.Z., Naeini S.A. (2012). Imrovement of loose sandy soil deposits using micropiles. KSCE J. Civ. Eng..

[35] Sun S., Zhu B., Ma H. (2010). Model experimental research on anti-sliding characteristics of micropiles with cap beam. Chin. J. Rock Mech. Eng..

[36] Lai H.J., Zheng J.J., Cui M.J., Chu J. (2020). “Soil arching” for piled embankments: Insights from stress redistribution behaviour of DEM modelling. Acta Geotech..

[37] Fang K., Miao M.H., Tang H.M., Dong A., Jia S.X., An P.J., Zhang B.C., Tu J.M. (2022). Model test on deformation and failure behaviour of arching-type slope under excavation condition. Eng. Geol..

[38] Xin J., Zheng Y., Tang X. (2014). Research on failure mechanism of anti-sliding micropiles Based on elastoplastic model. Chin. J. Rock Mech. Eng..

[39] Zeng J.X., Xiao S.G. (2020). A Simplified Analytical Method for Stabilizing Micropile Groups in Slope Engineering. Int. J. Civ. Eng..

[40] Hirai H. (2016). Analysis of piles subjected to lateral soil movements using a three-dimensional displacement approach. Int. J. Numer. Anal. Methods Geomech..

[41] Ashour M., Pilling P., Norris G. (2004). Lateral behavior of pile groups in layered soils. J. Geotech. Geoenvironmental Eng..

